# Age‐Related Characteristics of SYT1‐Associated Neurodevelopmental Disorder

**DOI:** 10.1002/acn3.70267

**Published:** 2025-12-02

**Authors:** Sam G. Norwitz, Josefine Eck, Joel S. Winston, Kate Baker

**Affiliations:** ^1^ MRC Cognition and Brain Sciences Unit University of Cambridge Cambridge UK; ^2^ Institute of Psychiatry, Psychology & Neuroscience King's College London London UK; ^3^ Department of Clinical Neurophysiology King's College Hospital NHS Foundation Trust London UK; ^4^ Department of Genomic Medicine University of Cambridge Cambridge UK; ^5^ Department of Pathology University of Cambridge Cambridge UK

**Keywords:** Baker‐Gordon syndrome, neurodevelopmental disorder, SYT1, SYT1‐associated neurodevelopmental disorder

## Abstract

**Objectives:**

We describe the clinical manifestations and developmental abilities of individuals with SYT1‐associated neurodevelopmental disorder (Baker‐Gordon syndrome) from infancy to adulthood. We further describe the neuroradiological and electrophysiological characteristics of the condition at different ages, and explore the associations between these characteristics and clinical symptoms.

**Methods:**

Participants were recruited to the UK‐based Brain and Behavior in Neurodevelopmental Disorders of Genetic Origin project. Caregivers completed a medical history questionnaire and a battery of standardized neurodevelopmental measures. MRI and EEG records were obtained with consent from treating clinicians. Age‐related clinical manifestations and neuroimaging records were systematically analyzed. Balanced accuracy testing was used to explore brain‐symptom associations.

**Results:**

This study describes 40 individuals with 30 distinct de novo *SYT1* variants, including 10 novel variants. Qualitative age‐related clinical trends included the resolution of hypotonia and worsening of movement disorders, sleep difficulties, and self‐injurious behaviors. Social‐communicative impairments were prominent, with evidence of progression with age. MRI abnormalities were identified in 45% of individuals, while EEG abnormalities were present in 93%. Epileptiform activity frequently co‐occurred with movement disorders, while irregular sleep EEG coincided with sleep difficulties and respiratory problems.

**Interpretation:**

This study characterizes the broad spectrum and age‐related progression of clinical symptoms and brain‐related findings in individuals with SYT1‐associated neurodevelopmental disorder. Further research is needed to understand factors contributing to within‐individual change, and to develop targeted interventions aimed at improving outcomes and quality of life for affected individuals and their families.

## 1 Introduction

Synaptotagmin 1 (SYT1) is an integral membrane protein that serves as a critical effector of presynaptic vesicle fusion, responsible for coupling activity‐induced calcium (Ca^2+^) influx to fast, synchronous neurotransmission within the central nervous system [1, 2]. SYT1 is comprised of two cytoplasmic functional domains (C2A and C2B) containing negatively charged aspartate residues that bind Ca^2+^ upon depolarization‐induced influx at the nerve terminal. Ca^2+^ binding neutralizes these residues and induces a conformational change, which allows the protein to penetrate the negatively charged plasma membrane, triggering vesicle fusion and release of neurotransmitters into the synaptic cleft [2, 3]. The *SYT1* gene is highly conserved across species, signaling its importance in neuronal function and neurodevelopment [3, 4].

De novo protein‐altering variants in the *SYT1* gene result in functional impairment of presynaptic vesicle exocytosis [5, 6, 7]. SYT1‐associated neurodevelopmental disorder (SYT1‐associated NDD; Baker‐Gordon syndrome; OMIM #618218), arising from these variants, is characterized by infantile hypotonia, ophthalmic abnormalities, moderate to profound global developmental delay, hyperkinetic and involuntary movement disorders, and EEG abnormalities in the absence of overt seizures in most cases [8]. With only 27 confirmed cases across the literature and no existing longitudinal or retrospective studies, there is limited information about the developmental trajectories and long‐term prognoses of diagnosed individuals. Moreover, no studies to date have attempted to systematically describe the neuroradiological and electrophysiological characteristics of the disorder, and relate these characteristics to clinical evolution.

Since the first reported case of SYT1‐associated NDD by our group a decade ago [5], the number of diagnosed individuals has increased considerably, and individuals previously reported have grown older. As such, we are now able to document symptom progression and neurobiological characteristics of SYT1‐associated NDD, and examine their associations, with a view towards better understanding this rare disorder and strengthening post‐diagnostic counseling and supportive care. In this paper, we retrospectively describe symptom manifestation and characterize the neuroradiological and electrophysiological features of the disorder across age bands from infancy through adulthood. We further explore the associations between neurobiological features and clinical symptoms.

## 2 Methods

### 2.1 Recruitment

Individuals with *SYT1* variants were recruited over a ten‐year period (2014–2024) to the UK‐based Brain and Behavior in Neurodevelopmental Disorders of Genetic Origin (BINGO) project. Study participants had received their genetic diagnosis within a local clinical service, following genome‐wide sequencing (WES or WGS, trio or solo) by an accredited laboratory. All variants were confirmed de novo. Families were referred to BINGO following diagnosis by clinicians, or families self‐referred via the project website. Written consent to share clinical and genetic data and collect phenotyping questionnaires for research purposes was obtained from parents or legal guardians under Cambridge Central Research Ethics Committee approval (IRAS 83633, REC ref.: 11/0330/EE). A systematic literature search was conducted to identify previously reported individuals with SYT1‐associated NDD who were not part of the BINGO cohort. This yielded five additional cases, all of whom met the inclusion criteria of a de novo protein‐altering (missense, indel, or insert) variant [9, 10, 11, 12, 13]. Published clinical histories were extracted from these reports and incorporated into the aggregate dataset where applicable.

### 2.2 Data Collection

Lifetime medical history information was collected from parents/carers via a study‐specific medical history questionnaire (MHQ) covering prenatal, infant, childhood and current health, as well as an inventory of developmental milestones. The MHQ was completed via an online form. For the five supplemental case reports, MHQ data were extracted from the published information where available. Parents/carers completed a battery of standardized neurodevelopmental questionnaires, with scoring and analysis procedures outlined in Table S1. All questionnaires were completed online except for Vineland Adaptive Behavior Scales (Vineland‐3) assessments [14], which were completed via video interview (*n* = 24) or telephone (*n* = 1), based on parent preference. For Vineland‐3 only, two individuals completed the assessment twice at different ages, in which case the most recent assessment was used for analysis. For parents/carers who did not have a working knowledge of English, a translator was provided by the research team and all questionnaires were completed via video interviews (*n* = 6). Translations were conducted in Spanish, Portuguese, Dutch, German, and Italian, with interpreters selected according to language availability. Translators were made familiar with the interview questions prior to the session and had the opportunity to discuss the questions with the researcher in advance to resolve any potential ambiguities in meaning. Clinical MRI (*n* = 58 reports from 31 individuals) and EEG (*n* = 65 reports and 6 recordings from 27 individuals) records were provided by clinicians and/or hospitals with parent/carer consent.

### 2.3 Data Analysis

Data were analyzed using MATLAB, version R2023b and R, version 4.4.2. Retrospective MHQ data was organized by age band (infancy [< 1 year], childhood [1–10 years], and adolescence to adulthood [> 10 years]). Where participants had partial datasets, all available data were included in analyses, in line with Martin et al (2025) [15]. Bar graphs were used to display symptom prevalence for each age band. Growth parameters were calculated in accordance with CDC growth chart percentile curves [16].

Raincloud plots were generated for each questionnaire measure as previously described [15]. Where applicable, cut‐off scores were overlaid to contextualize the severity of difficulties relative to normative populations or previously published data. Spearman's correlation was used to assess for a monotonic relationship between questionnaire scores and age, as a non‐parametric method appropriate for our relatively small cohort. Results were visualized using a forest plot.

Kaplan–Meier survival analyses were used to model the ages at which participants achieved core developmental milestones. Analyses included participants who had either achieved the milestone or who were of an age when achieving each milestone could be expected, with lower bounds of inclusion based on the age at which 50% of typically developing children pass the milestone [17, 18].

MRI reports were collated and grouped by age band as above. Reports were classified as either (1) normal or as reporting (2) developmental abnormalities and/or (3) acquired abnormalities, based on the classification schemes described by Ashwal et al. [19] and Robinson et al. [20]. The latter two categories consisted of eight primary patterns of abnormality, including: (i) irregular skull morphology, (ii) delayed or immature myelination, (iii) cysts and masses, (iv) hypoplasia, (v) heterotopia, (vi) white matter lesions or abnormalities, (vii) cortical/subcortical atrophy, and (viii) hemorrhagic lesions. The determination of abnormalities was made by the treating clinicians and/or radiologists who interpreted the MRI scans and authored the reports. For individuals with multiple MRIs, serial longitudinal findings were compared to identify changes over time.

EEG records were collated and grouped by age band. All available reports and recordings were reviewed in consultation with a clinical neurophysiologist and classified according to the status of the following criteria: (i) overall abnormal, (ii) abnormal posterior background, (iii) epileptiform activity (wakeful and sleep‐state), (iv) non‐specific abnormalities (wakeful and sleep‐state), (v) abnormal sleep features/architecture, (vi) superimposed abnormalities in sleep, (vii) abnormal response to photic stimulation, and (viii) events captured. Non‐specific abnormalities were defined as irregular patterns of activity that were not characteristic of a particular etiology, and were classified as focal, multifocal, or generalized. Events were defined as clinically observed behaviors captured on EEG and were classified as epileptic or not. Reports and recordings with insufficient data or descriptions to allow for confident overall categorization were excluded from the final analysis. For individuals with multiple EEGs, serial findings were compared to identify changes over time.

To explore brain‐symptom associations, we conducted a data‐driven exploratory analysis using balanced accuracy testing to identify concurrent patterns of co‐occurrence between neuroimaging features and clinical symptoms. Balanced accuracy was calculated as the average of sensitivity and specificity to account for class imbalance within the dataset. A score of 0.5 indicates performance equivalent to chance, while higher scores reflect a stronger‐than‐chance co‐occurrence. To account for repeated measures from participants with serial scans, a bootstrapping approach was employed, in which a random subset of scans was selected for analysis, ensuring that only one observation per participant was included in each iteration. This process was repeated 500 times, and balanced accuracy values were averaged across iterations to generate stable estimates that accounted for variability in participant selection while minimizing overfitting to a specific subset of data. The results were visualized using a heatmap displaying classification performance across feature pairs.

## 3 Results

### 3.1 Participants and Genetic Diagnoses

Forty‐two individuals with *SYT1* gene variants were recruited to the BINGO project. Inclusion criteria for the current study included a confirmed de novo protein‐altering (missense, indel, or insert; non‐truncating) *SYT1* variant, without an alternative candidate variant more likely to explain the individual's neurodevelopmental presentation. Based on these criteria, seven individuals were excluded from the analysis (four with truncating variants, one with an inherited likely non‐pathogenic variant, one with a homozygous variant, and one with an alternative pathogenic variant), because of a lack of current evidence that these classes of variant can be pathogenic. An additional five study subjects who met the inclusion criteria were identified from published case reports [9, 10, 11, 12, 13]. Thus, a total of 40 individuals with 30 distinct variants were included in the final analysis. See Table S2 for gene variant information and pathogenicity classifications (compiled using the Pathogenicity Evidence tool on Decipher, according to the American College of Medical Genetics and Genomics [ACMG] 2015 criteria) [21, 22].

### 3.2 Physical and Behavioral Symptoms

At the time of assessment, participants included 2 (5%) infants, 24 (60%) children, and 14 (35%) adolescents and/or adults (Table 1). Retrospective data were available for all applicable prior age bands for every individual. Thus, data were available for a total of 40 individuals from infancy, 38 from childhood, and 14 from adolescence and/or adulthood.

**TABLE 1:** Participant demographics.

Demographics	*N* data available	Mean	Range	SD
Age at time of participation, years	40 (100%)	9.1	0.2–34.3	7.7
Infant (0–1 year)	2 (5%)	0.5	0.2–0.9	0.5
Child (1–10 years)	24 (60%)	5	1.2–8.4	2.2
Adolescent (> 10 years)	14 (35%)	17.5	10.8–34.3	7.1
Sex (female, no.)	19 (47.5%)			
Deceased (after assessment), years	3 (7.5%)	7.4	0.2–11	6.2
Pregnancy	37 (92.5%)			
Pregnancy length, weeks	34/37 (91.9%)	39.1	34–42.5	2
Pregnancy concerns	19/35 (54.3%)			
Reduced fetal movements	8/35 (22.9%)			
NICU/SCBU admission	8/30 (26.7%)			
Growth parameters				
Birth weight, kg	36 (90%)	3.2	2.2–4.2	0.6
Current weight, percentile	23 (57.5%)	22.8	0.4–97.8	28.9
Current height, percentile	22 (55%)	31.9	0.9–99.8	31.7
Orbitofrontal circumference, percentile	23 (57.5%)	33.2	0.6–99	30.6

### 3.3 Abnormal Muscle Tone

Hypotonia was highly prevalent in infancy (38/40 [95%]) and childhood (32/36 [88.9%]), but reduced in adolescence and adulthood (5/12 [41.7%]). A concomitant increase in hypertonia/stiffness was observed in six children and two adolescents, though the overall prevalence of hypertonia remained low (< 20%) across age bands.

### 3.4 Movement Disorders

**FIGURE 1:**
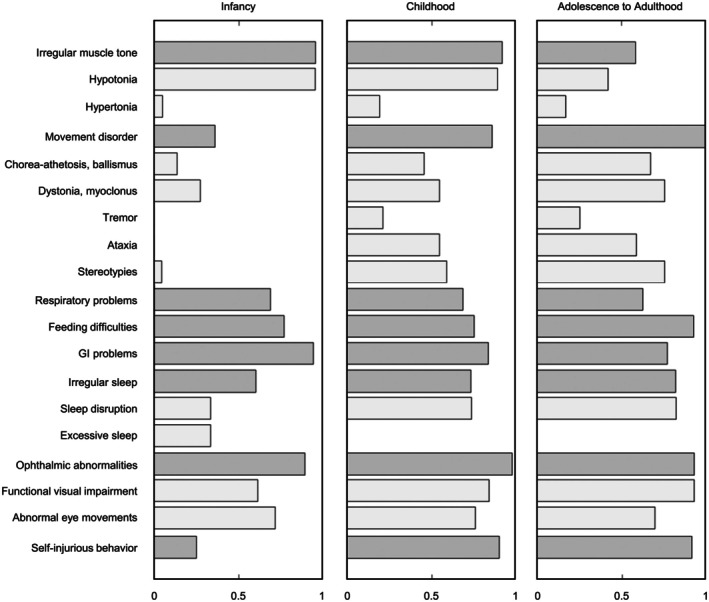
Clinical symptom prevalence by age band. Bar graphs depict the fraction of individuals across age bands who display each symptom. Age bands include infancy (< 1 year), childhood (1–10 years), and adolescence to adulthood (> 10 years). Dark gray bars represent the overall prevalence for each symptom category; light gray bars represent subtype prevalence, if applicable.

The overall prevalence of movement disorders increased from 36.4% (8/22) in infancy to 85.7% (30/35) in childhood, and further increased to 100% (13/13) in adolescence and adulthood. Figure 1 shows a breakdown of movement disorder subtypes by age band. Hyperkinetic movement disorders were the most common across age bands, with little change in distribution from childhood onwards. Ataxia first emerged in childhood and persisted into adolescence/adulthood.

### 3.5 Respiratory Problems

Respiratory problems were common from birth through adulthood without considerable change in overall prevalence. The most common respiratory problem was apnea, with aspiration, irregular breathing patterns and recurrent respiratory infections also common. Of the eight newborns who required NICU/SCBU admission (Table 1), seven were due to respiratory difficulties. One newborn died at 57 days of age due to respiratory complications [10].

### 3.6 Feeding and Gastrointestinal (GI) Problems

Mechanical feeding problems, such as chewing, swallowing and sucking difficulties, were common without considerable change across age bands. Nine individuals required surgical procedures to assist with enteral feeding. Accompanying these feeding difficulties was a high prevalence of GI problems, which persisted across age bands. The most common GI problem in infants was gastroesophageal reflux disease, with irregular bowel motility also being common from childhood onwards.

### 3.7 Sleep Problems

In infancy, sleeping patterns varied widely, with 33.3% (10/30) reported to sleep excessively and 33.3% (10/30) having difficulties commencing and staying asleep. A substantial increase in sleep disruptions was observed in childhood (73.3% [22/30]), including the emergence of sleep disruptions in all the individuals who previously slept excessively, which persisted into adolescence and adulthood (81.8% [9/11]). Typical sleep disruptions were characterized by difficulty commencing sleep, frequent waking throughout the night, nighttime agitation, and extended periods without sleep.

### 3.8 Ophthalmic Abnormalities

Functional visual impairments and abnormal eye movements were highly prevalent across age bands. The most common functional impairment was CVI, present in 22.2% (8/36) of infants, 48.6% (18/37) of children, and 69.2% (9/13) of adolescents and adults. Issues with visual acuity, including hypermetropia and myopia, were also common, present in 27.8% (10/36) of infants, 51.4% (19/37) of children, and 46.2% (6/13) of adolescents and adults. The most common abnormal eye movement was strabismus (esotropia, exotropia), present in 50% (18/36) of infants, 56.8% (21/37) of children, and 53.8% (7/13) of adolescents and adults. Nystagmus was also common, present in 41.7% (15/36) of infants, 45.9% (17/37) of children, and 15.4% (2/13) of adolescents and adults.

### 3.9 Self‐Injurious Behavior

Self‐injurious behaviors—defined as repetitive, harmful actions directed towards one's own body—were reported in only two infants (25%) but became highly prevalent in childhood (26/29 [89.7%]) and persisted into adolescence and adulthood (90.9% [10/11]). The most common form of self‐injurious behavior was chewing one's body parts (typically fingers and hands), followed by self‐hitting, biting and head banging.

### 3.10 Developmental Milestones

**FIGURE 2:**
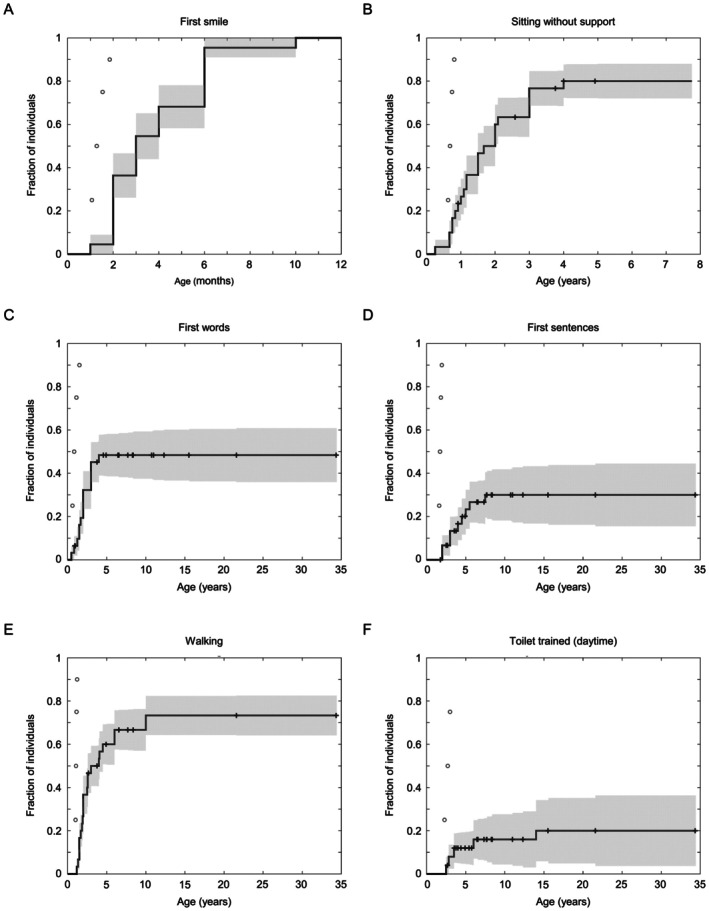
Acquisition of developmental milestones. Kaplan–Meier plots depict the fraction of participants who have achieved each milestone: (A) First smile; (B) Sitting without support; (C) First words; (D) First sentences; (E) Walking; and (F) Toilet trained (daytime). Individuals who are of an appropriate age but who have yet to achieve each milestone are indicated by crosses with their age at the time of assessment. Normative percentiles for milestone acquisition are displayed as open circles [14, 16].

The chronological ages at which individuals achieved developmental milestones are summarized in Figure 2. In all instances, the age of reaching developmental milestones was delayed when benchmarked to normative percentiles [17, 18]. Smiling emerged in all individuals, at a median age of 3 months. Sitting unsupported was attained by 80% (24/30; lower bound of inclusio*n* = 8.2 months) at a median age of 18 months. Speaking in single words occurred in 48.4% (15/31; lower bound of inclusio*n* = 10.1 months), with a median age of 2 years, while 30% (9/30; lower bound of inclusio*n* = 20.6 months) progressed to speaking in sentences, with a median age of 4 years. Walking was achieved by 73.3% (22/30; lower bound of inclusion = 12.9 months) at a median age of 2.3 years. Daytime toilet training was achieved by 20% (5/25; lower bound of inclusio*n* = 31.5 months for girls, 34.7 months for boys) at a median age of 3.5 years.

### 3.11 Neurodevelopmental Questionnaires

**FIGURE 3:**
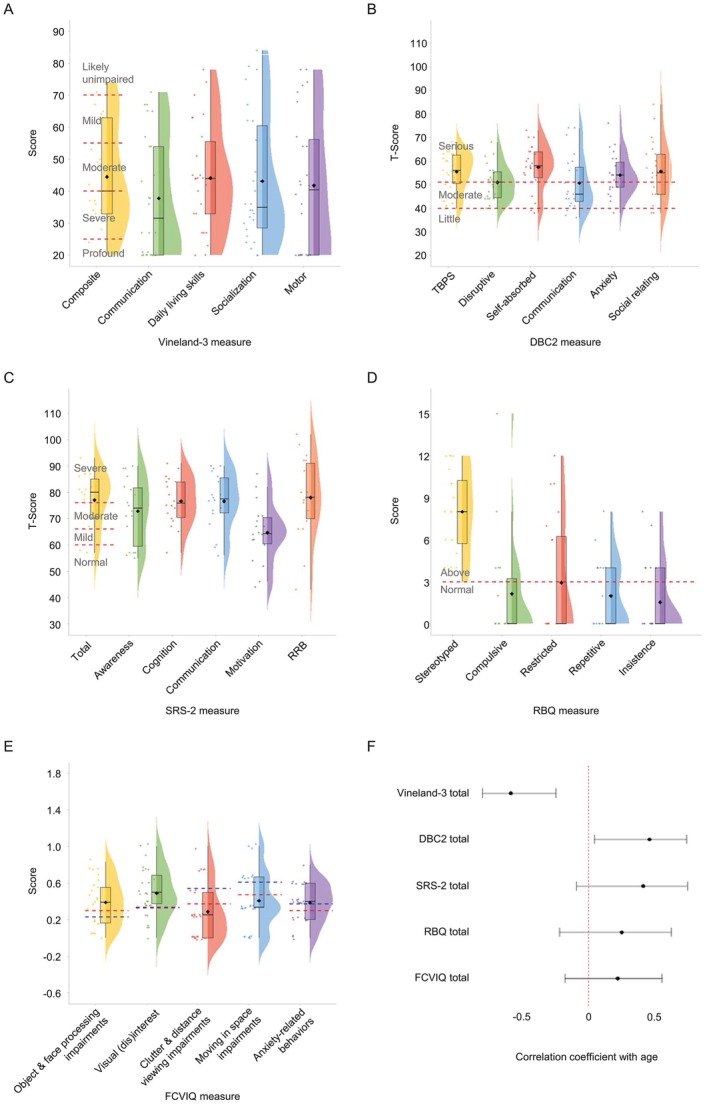
Neurodevelopmental questionnaire measure scores. Violin plots display the distribution of scores on each of the questionnaire measures. Mean scores on each measure are represented by a black diamond. (A) Age‐standardized Vineland Adaptive Behavior Scales (Vineland‐3). ID severity cut‐offs are overlaid in red for the Vineland‐3 composite score. (B) Developmental Behavior Checklist (DBC2) T‐scores. Levels of concern are overlaid in red for DBC2 subscales. (C) Social Responsiveness Scale (SRS‐2) T‐scores. Severity cut‐offs are overlaid in red for the SRS‐2 total score. (D) Restricted and Repetitive Behavior Questionnaire (RBQ) scores. Severity cut‐offs are overlaid in red for all RBQ subscales. (E) Flemish Cerebral Visual Impairment Questionnaire (FCVIQ) factors scores. For reference, mean scores for children with CVI are overlaid in red [23], and mean scores for children with unilateral cerebral palsy and co‐occurring CVI are overlaid in blue [24]. (F) Forest plot displaying Spearman correlation coefficients and 95% confidence intervals for the association between age and questionnaire measures.

Vineland‐3 Composite scores indicated a wide range of adaptive abilities, with 12% (3/25) of individuals falling within the normal/borderline range, 40% (10/25) classified as mildly to moderately impaired, and 48% (12/25) classified as severely to profoundly impaired. Among the Vineland‐3 subdomains, communication was the most affected (Figure 3A).

The majority of Developmental Behavior Checklist (DBC2) [25] scores indexing emotional and behavioral difficulties were in the moderate to serious concerns range, with relatively greater difficulties with self‐absorbed behavior—a DBC‐2 subscale involving diverse items including mouthing objects, special interests and stereotyped behaviors (Figure 3B).

Social Responsiveness Scale (SRS‐2) [26] scores indicated high levels of autism‐associated traits, with scores spanning the mild‐to‐severe range of atypicality. Social motivation—an index of a child's interest in social interaction and desire to engage with peers and in shared activities—emerged as a relative strength compared to other SRS‐2 subscales (Figure 3C).

The Repetitive Behavior Questionnaire (RBQ) [27] assesses five domains of restrictive and repetitive behaviors. Most RBQ subdomains were within the normal threshold, except for stereotyped behaviors, which were markedly elevated (Figure 3D).

The Flemish Cerebral Visual Impairment Questionnaire (FCVIQ) [28] is a measure of behaviors associated with cerebral visual impairment (CVI) and its impact on everyday functioning. FCVIQ sum scores identified 81.5% (22/27) of individuals to be at risk for CVI (M = 4.7, SD = 1.3, factor sum range: 2–6). Factor scores on the FCVIQ revealed difficulties with object and face processing, visual interest, and anxiety‐related behaviors of a level comparable to children with CVI [23, 24] (Figure 3E).

Spearman correlation analysis revealed a significant negative association between participant age at the time of data collection and Vineland‐3 composite scores (*r*_s_ = −0.58, *n* = 25, *p* = 0.002, 95% CI [−0.80, −0.24]), indicating lower global adaptive functioning in older individuals within the group. Additionally, a significant positive association was found between age and DBC scores (*r*_s_ = 0.46, *n* = 22, *p* = 0.03, 95% CI [0.044, 0.74]), indicating more frequent or severe behavioral and emotional challenges in older individuals (Figure 3F).

### 3.12 Neuroradiology and Electrophysiology

A total of 58 MRI reports were obtained from 31 individuals. This included 25 scans from 21 infants, 24 scans from 19 children, and 10 scans from 6 adolescents and adults. Fifteen individuals had serial MRI scans across multiple age bands, enabling observation of within‐participant change. The findings are summarized in Table 2. Sample MRI images are provided in Figure S1.

**TABLE 2:** MRI classifications by age band.

MRI classification	Infants (*n* = 21 participants, *n* = 25 scans)	Children (*n* = 19 participants, *n* = 24 scans)	Adolescents and adults (*n* = 6 participants, *n* = 10 scans)	Total, MRI feature ever reported (*n* = 31 participants, *n* = 58 scans)
Normal	12 (57.1%)	13 (68.4%)	3 (50%)	17 (54.8%)
Developmental abnormalities and malformations	8 (38.1%)	6 (31.6%)	3 (50%)	13 (41.9%)
Irregular skull morphology	6	4	1	8
Delayed/immature myelination	4	1	0	4
Cysts and masses	2	1	1	4
Hypoplasia	3	2	1	4
Heterotopia	0	1	1	2
Acquired lesions	2 (9.5%)	4 (21.1%)	2 (33.3%)	7 (22.6%)
White matter lesions/abnormalities	1	3	1	4
Cortical/subcortical atrophy	—	3	2	5
Hemorrhagic lesions	1	0	0	1

*Note:* Values indicate the number of individuals in each age band who display the feature. For each feature, the denominator represents the total number of individuals for whom that feature was classifiable based on available information. Values in the ‘total’ row indicate the total number of individuals who display the respective feature across age bands, accounting for individuals who are represented in multiple age bands.

The most prevalent developmental malformation was irregular skull morphology, most commonly microcephaly. Delayed myelination was common in infancy but typically resolved by childhood. Structural abnormalities included two left temporal arachnoid cysts, two non‐specific focal masses, and hypoplasias of the septo‐optic/pituitary region, hippocampal‐fornix system, corpus callosum, insula, operculum, and posterior fossa. Acquired lesions consisted of persistent periventricular white matter lesions, and a choroid plexus hemorrhage in one infant which resolved in childhood. Progressive atrophy was observed for five individuals over serial scans and involved focal cerebellar and corpus callosal atrophy as well as three instances of diffuse cortico‐subcortical atrophy with secondary ventricular dilation.

A total of 70 EEG reports and 6 recordings were obtained from 31 individuals. Upon review by a consultant neurophysiologist, 65 reports and 6 recordings from 27 patients had sufficient data for overall classification and between‐subject comparison. This included 18 records from 13 infants, 39 records from 20 children, and 8 records from 6 adolescents and adults. The findings are summarized in Table 3. Sample EEG images are provided in Figure S2.

**TABLE 3:** EEG classification by age band.

EEG classification	Infants (*n* = 13 participants, *n* = 18 records)	Children (*n* = 20 participants, *n* = 39 records)	Adolescents and adults (*n* = 6 participants, *n* = 8 records)	Total, EEG feature ever‐reported (*n* = 27 participants, *n* = 65 records)
Wakeful scans captured	12 (92.3%)	20 (100%)	6 (100%)	27 (100%)
Overall abnormal	11/12	19/20	5/6	25/27
Abnormal posterior background	7/8	12/14	3/5	17/22
Epileptiform activity	5/9	14/19	4/6	19/27
Non‐specific abnormalities	10/10	15/16	4/5	21/23
Sleep scans captured	4 (30.8%)	9 (45%)	2 (33.3%)	13 (48.1%)
Abnormal sleep features	1/3	2/7	0/2	3/10
Superimposed abnormalities	4/4	9/9	1/2	12/13
Epileptiform activity	2/4	8/9	1/2	9/13
Non‐specific abnormalities	4/4	5/7	1/2	8/10
Abnormal photic stimulation	1/4 (25.0%)	1/6 (16.7%)	0/3 (0%)	2/12 (16.7%)
Events captured	3*	3	0	4

*Note:* Values indicate the number of individuals in each age band who display the feature. For each feature, the denominator represents the total number of individuals for whom that feature was classifiable based on available information. Values in the ‘total’ row indicate the total number of individuals who ever display the respective feature across age bands, accounting for individuals who are represented in multiple age bands.

* One epileptic event classified as seizure‐type flexor spasm (captured in Figure S2E).

Wakeful states were captured across 63 studies for 27 individuals. The archetypal wakeful pattern consisted of abnormal posterior background rhythms, primarily characterized by bursts of synchronous high‐voltage polymorphic slow waves. Focal and/or multifocal epileptiform activity was commonly observed (19/27 [70.4%]), with a tendency towards maximal activity in bilateral posterior regions. Importantly, such epileptiform activity was evident without any associated behavioral manifestations suggesting a clinical seizure. Sleep was captured in 27 recordings across 13 individuals. Physiological sleep architecture was largely normal. Epileptiform activity, characterized by high amplitude spike–wave complexes and sharp waves, enhanced in sleep and tended to increase and spread with slow wave sleep. Of the 4 individuals without observed epileptiform abnormalities, 3 displayed non‐specific abnormalities during sleep, with both focal and generalized localization. Where commented upon, abnormal paroxysmal patterns on sleep EEG were not associated with observations suggestive of clinical seizure.

Eleven individuals had serial EEGs across multiple age bands. Of these, EEGs remained overall abnormal for 10 individuals and became abnormal for one. Wakeful epileptiform activity emerged in three individuals and persisted in six. Two individuals had serial sleep studies, both of which remained abnormal with epileptiform activity and superimposed abnormalities.

### 3.13 Clinical and Neurobiological Correlates

**FIGURE 4:**
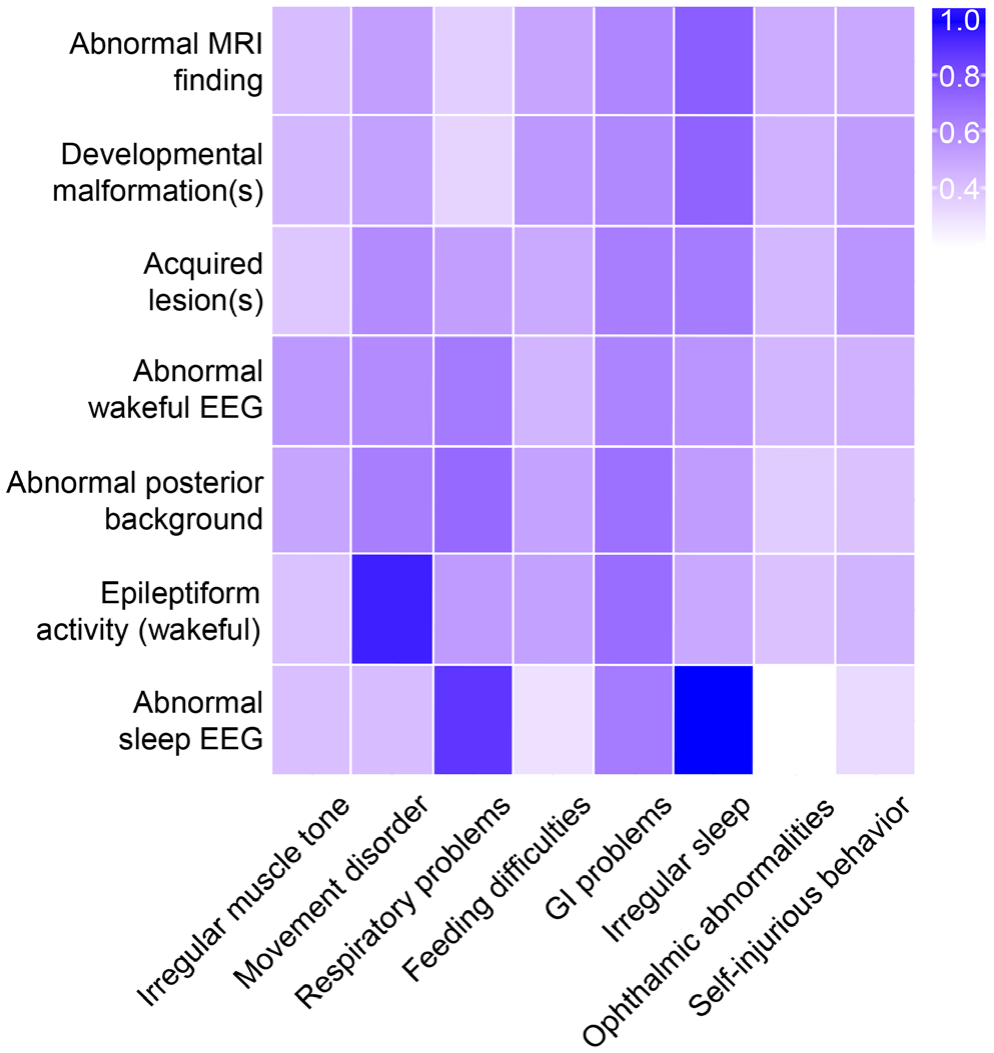
Balanced accuracy heatmap for neuroimaging features and clinical symptoms. Heatmap depicting balanced accuracy for EEG/MRI features paired with concurrent clinical symptoms. Darker shades indicate higher classification accuracy. [Correction added on 14 February 2026, after first online publication: Figure 4 has been replaced.]

Balanced accuracy testing of all available cross‐sectional data—with bootstrapping to account for multiple observations per individual across age bands—revealed that overall abnormal MRI findings were concurrent with clinical features with an average balanced accuracy of 52%, and abnormal wakeful and sleep EEG findings had average balanced accuracies of 54% and 53%, respectively (Figure 4). That is, neuroimaging and neurophysiology findings aligned with clinical symptom presence and absence in approximately half of observations (the level expected by chance). A few brain–symptom pairs, however, demonstrated notably high performance. Epileptiform activity co‐occurred with movement disorders with 95% balanced accuracy, while abnormal sleep EEG findings were fully concordant with irregular sleep patterns (100%) and showed high concordance with respiratory problems (88%).

## 4 Discussion

In this study, we retrospectively mapped age‐related physical and behavioral symptoms in 40 individuals with SYT1‐associated NDD. We further analyzed 123 MRI and EEG records across 32 individuals, assessing age‐related characteristics and relation to clinical presentation. The clinical features associated with *SYT1* variants in the current study are consistent with previous smaller case series [8]; however, age‐related trends and associations with neurobiological characteristics can now be observed for the first time.

Age‐band analysis indicated that hypotonia often resolves with age, and, in some cases, is associated with a concomitant increase in hypertonia and spasticity. Similar dynamic shifts in muscle tone have been observed in infantile hypotonia of heterogeneous origins, with contributions from genetic disorders affecting key structural proteins of the nervous system [29]. Since muscle tone is essential not only for gross motor development but also for smooth muscle function, is it conceivable that hypotonia may be a contributing factor to respiratory distress and feeding difficulties in infants and children with *SYT1* variants [29, 30].

Another age‐related trend was the increasing prevalence of involuntary movement disorders, primarily of the hyperkinetic type. Given that SYT1 is the primary isoform facilitating terminal dopamine release in the midbrain, its dysfunction likely contributes to the predominance of basal ganglia‐related movement disorders, including chorea‐athetosis, ballismus, dystonia, myoclonus, and stereotypies [2, 31]. The progression of motor symptoms may reflect a combination of mechanisms, including progressive synaptic dysfunction, maladaptive plasticity and reorganization, and the failure of compensatory mechanisms within the cortico‐basal ganglia‐thalamic circuitry [32]. However, we also observed considerable variation in the types and severity of movement abnormalities within the group, for reasons that are not yet understood. It is important to consider that this apparent progression with age may be influenced by ascertainment bias—as these features may prompt genetic testing—and by under‐recognition in younger or non‐ambulatory children. Clarifying whether these trends reflect true biological progression, such as progressive synaptic dysfunction or age‐related neuronal loss, will thus be an important goal for future studies.

Retrospective assessment of sleep habits revealed a developmental pattern characterized by excessive sleepiness in infancy followed by the emergence of severe sleep disturbances in childhood, with frequent waking and extended periods without sleep (sometimes lasting days). Sleep disorders are highly prevalent in individuals with pervasive NDDs, affecting up to 86% of cases [33, 34]. Potential contributors include hormonal imbalances, neurotransmitter dysregulation, medical comorbidities (e.g., respiratory issues, GI dysfunction, epilepsy), medication effects, sensory sensitivities and environmental stressors [33]. Sleep disturbances in NDDs are also strongly linked to behavioral and psychological factors, including lower adaptive function, increased internalizing and externalizing behaviors and greater autism symptom severity [35]. The relationship is bidirectional, as children with NDDs may be more vulnerable to sleep disturbances due to underlying biological and behavioral factors, while chronic sleep deprivation exacerbates neurodevelopmental challenges [35, 36, 37, 38]. In individuals with SYT1‐associated NDD, the evolution of sleep problems may reflect a convergence of changes in medical comorbidities (e.g., respiratory and gastrointestinal dysfunction, epileptiform activity), medication use, and the progression of cognitive and behavioral differences, all of which likely contribute to the observed developmental shift from excessive sleepiness in infancy to disrupted sleep patterns in childhood and adolescence.

In line with our previous research, we found considerable variability in the acquisition of developmental milestones and adaptive functioning, with most individuals showing mild to profound impairments, particularly in communication [8]. Building on these findings, standardized questionnaire measures highlight variably severe social–emotional–behavioral concerns including pronounced self‐absorbed behaviors, anxiety, and autism‐related traits. Stereotyped behaviors, self‐injurious behaviors, and poor functional vision (CVI) were also reported to be prevalent within the SYT1 group, highlighting the challenge of discriminating between motor or sensory problems versus cognitive or behavioral characteristics. Cross‐sectional analysis highlighted the potential for worsening adaptive functions with age; however again this should be interpreted with caution because of the differences in ascertainment for genome‐wide testing over time (i.e., younger‐diagnosed individuals may be from a less severely impacted population on average) and previously observed decline in scores on age‐standardized measures within NDD populations [39].

Whereas previous reports of SYT1‐associated NDD have described predominantly normal MRI findings [6, 8, 9, 10], the current study found that approximately half of individuals had some atypical observations reported on their MRI. These heterogeneous observations may be incidental or of limited functional significance and tended to stabilize or resolve with age. In some individuals, however, serial scans were reported to show evidence of progressive cortico‐subcortical atrophy. Systematic longitudinal imaging will be essential to clarify whether such progressive changes represent a consistent feature of the disorder and reflect clinically relevant pathology.

In line with prior reports [6, 8, 9], EEGs collated for this SYT1‐associated NDD cohort consistently displayed widespread abnormalities. These were characterized by delayed background activity interspersed with bursts of synchronous high‐voltage slow waves, predominantly in posterior regions, alongside isolated epileptiform discharges. In this study, we found largely preserved sleep architecture but a high prevalence of epileptiform activity, exacerbated by slow‐wave sleep. Analysis of EEG features over time highlighted a general increase in abnormalities, whilst cross‐correlating EEG and clinical characteristics highlighted co‐occurrence between EEG abnormalities and movement disorders, respiratory problems and sleep disruptions with high balanced accuracies. Epileptiform activity and movement disorders frequently co‐occur in genetic neurodevelopmental conditions, with presentation patterns and temporal relationships varying by underlying genetic etiology [40, 41, 42]. Likewise, the relationship between sleep disruption and epileptiform activity is well documented across a range of etiologies, with non‐REM slow‐wave sleep potentiating epileptiform discharges and, in turn, interictal activity fragmenting sleep architecture [43, 44]. An established dynamic interplay exists between these linked features and respiratory dysfunction. Sleep‐disordered breathing—particularly apnea, the most common respiratory symptom observed in this study—exacerbates discharge frequency and severity, and conversely, can be influenced by epileptic activity [45, 46].

Notably, in SYT1‐associated NDD cases, overt behavioral manifestations or clinical seizures have very rarely been observed [6, 8], despite the presence of epileptiform discharges during both wakefulness and sleep. This differentiates the condition from most other synaptic vesicle cycling disorders [47] and SNAREopathies [48]. The neuronal origins, functional significance and clinical implications of these EEG observations remain an important area for future research, as subclinical epileptiform activity has been linked to cognitive impairment and decline across various neurological and psychiatric disorders [49, 50]. It is plausible that the heightened level of cortical hyperexcitability and hypersynchronization of neuronal networks associated with EEG oscillations and epileptic activity could underlie impaired gating and modulation of motor commands, regulation of autonomic functions such as respiratory rate and sleep cycles, and neural systems functions relevant to cognition. Together, these findings underscore the need for deeper mechanistic understandings of the multisystem interactions in SYT1‐associated NDD to inform more precise clinical strategies for managing symptoms and promoting adaptive outcomes.

The current study has several limitations. First, we restricted our analysis to missense, indel, or insert variants, excluding truncating variants to align with the current mechanistic understanding of the disorder [2, 7]. While all variants were systematically assessed using ACMG criteria, the pathogenicity of 2/30 variants remains uncertain pending functional analysis and/or identification of further variants at these loci, mirroring the clinical reality of classification limitations for many ultra‐rare conditions. The effects of variant type, location and functional impact on clinical evolution over time should be a future priority topic. Second, given that each participant was diagnosed and recruited at a different age, some individuals were missing information from prior age bands. Third, as with many global rare disease studies, conducting standardized assessments across languages and cultures can be challenging, underscoring the importance of working with trained interpreters and using measures with established cross‐language and cross‐cultural comparability. Fourth, data for the five individuals who were identified through published case reports were not standardized. Fifth, multiple clinicians contributed to MRI and EEG reporting, which may have introduced variability and dataset inconsistencies. Sixth, while balanced accuracy testing provided a preliminary exploration of brain–symptom co‐occurrence in our small sample size, its descriptive nature precludes causal interpretation, as associations may be confounded by other factors. Lastly, SYT1‐associated NDD is a rare syndrome with approximately only 50 confirmed cases worldwide. It is possible that milder or less typical forms of the disorder remain undiagnosed, as ascertainment for genomic testing varies globally and continues to change over time. As more clinicians become aware of the syndrome, and whole genome sequencing becomes more widely available, more cases will be identified, and different clinical patterns may become apparent.

In conclusion, the current study presents the most comprehensive overview to date of the clinical, behavioral, neuroradiological, and electrophysiological features of SYT1‐associated NDD. We expanded the catalog of de novo *SYT1* variants and characterized the broad spectrum of clinical symptomatology and neuroimaging features in individuals with the disorder, including how they change over time from infancy through adulthood. Further research is needed to deepen our understanding of these developmental changes and to develop targeted interventions aimed at improving outcomes and quality of life for affected individuals and their families.

## Author Contributions

S.G.N. and K.B. contributed to the conception and design of the study. S.G.N. and J.E. contributed to the acquisition of clinical data; J.S.W. and S.G.N. contributed to the review of neuroimaging/EEG records. S.G.N. contributed to data analysis. S.G.N. and K.B. contributed to drafting the text and figures, and all authors revised and approved the submitted manuscript.

## Funding

This work was supported by the Medical Research Council (MC_UU_00030/3), Great Ormond Street Hospital Charity, NIHR Cambridge Biomedical Research Centre, and Gates Cambridge Trust.

## Conflicts of Interest

The authors declare no conflicts of interest.

## Supporting information


**Figure S1:** Sample MRI images.
**Figure S2:** Sample EEG images.
**Table S1:** Neurodevelopmental questionnaire measures.
**Table S2:** SYT1 gene variant information.

## Data Availability

Reporting of *SYT1* variants in open access repositories is listed in Table S2. Anonymized data may be made available to other researchers from the corresponding author on reasonable request, and on the condition of signing a Code of Conduct guaranteeing that the data will be kept confidential and secure.
