# Supplementary material for: Age‐Related Characteristics of SYT1‐Associated Neurodevelopmental Disorder
Source: Ann Clin Transl Neurol. 2025 Dec 2;13(5):875–87. doi: 10.1002/acn3.70267 (PMC13161886; doi:10.1002/acn3.70267)
Supplement: Supplementary file 1 — Figure S1: Sample MRI images. Figure S2: Sample EEG images. Table S1: Neurodevelopmental questionnaire measures. Table S2: SYT1 gene variant information. [file ACN3-13-875-s001.docx]

# Supplementary Materials for: Age-Related Characteristics of SYT1-Associated Neurodevelopmental Disorder

Sam G. Norwitz ^1, 2^, Josefine Eck ^1^, Joel S. Winston ^2, 3^, Kate Baker ^1, 4, 5^

*1: MRC Cognition and Brain Sciences Unit, University of Cambridge, United Kingdom*

*2:* *Institute of Psychiatry, Psychology & Neuroscience, King's College London, London, United Kingdom*

*3:* *King's College Hospital NHS Foundation Trust, London, United Kingdom*

*4: Department of Medical Genetics, University of Cambridge, United Kingdom*

*5: Department of Pathology, University of Cambridge, United Kingdom*

Contents

[**Supplementary Table 1.** Neurodevelopmental questionnaire measures](#_Toc177641311) 2

[**Supplementary Table 2.** SYT1 gene variant information](#_Toc177641311) 5

[**Supplementary Figure 1.** Sample MRI images](#_Toc177641313) 8

[**Supplementary Figure 2.** Sample EEG images](#_Toc177641313) 9

[**References** 1](#_Toc177641320)2

## **Supplementary Table 1.** Neurodevelopmental questionnaire measures.

| Questionnaire | Description and administration | Domains assessed | Scoring procedure | Scoring interpretation | Scoring categories |
| --- | --- | --- | --- | --- | --- |
| Medical History Questionnaire (MHQ) | Collects medical information during infancy, childhood and current (within the past six months). | - Muscle tone - Movement disorders - Feeding difficulties - Sleep difficulties - Sensory impairments - Psychiatric diagnoses - Self-injurious behaviors | Symptom presence or absence (with option for free text information) | Data represent raw prevalence counts | No predefined normal range or clinical cutoff. |
| The Vineland Adaptive Behavior Scales (Third Edition, Vineland-3) ^1^ | A standardized assessment of adaptive abilities.  *The Vineland-3 Parent/Caregiver form was administered via interview for internal comparability with the broader BINGO research studies and to overcome logistical and legal data protection constraints inherent to our global cohort.* | - Adaptive behavior composite - Communication - Daily living skills - Socialization - Motor skills | Raw scores were scaled according to published norms.  Motor skill scores for participants >= 10 years were calculated using the highest available age-band. | Lower scores indicate greater deficits in adaptive functioning compared to same-age peers. | Composite score severity categorized as ‘likely unimpaired’ (≥ 70), ‘mild’ (55-69), ‘moderate’ (40-54), ‘severe’ (25-39), or ‘profound’ (< 25). |
| Developmental Behavior Checklist (DBC2) ^2^ | A standardized measure of emotional-behavioral difficulties in individuals with ID | - Total problem behaviors - Disruptive/antisocial behavior - Self-absorbed behavior - Communication disturbance - Anxiety - Social relating | T-scores were generated using the Australian norms. | Higher T-scores indicate more severe or frequent problems. | T-scores < 40 indicate little concern, 40-50 indicate moderate concern, and ≥ 51 indicate serious concern. |
| Social Responsiveness Scale (SRS-2) ^3^ | A screening tool to assess autism characteristics. | - Total - Social awareness - Social cognition - Social communication - Social motivation - Restricted interests and repetitive behavior | Raw scores converted to age- and sex-appropriate T-scores based on published norms. | Higher T-scores indicate a greater impairment. | Total and subscale T-scores categorized as ‘normal’ (< 60), ‘mild’ (60-65), ‘moderate’ (66-75), or ‘severe’ (≥ 76). |
| Repetitive Behavior Questionnaire (RBQ) ^4^ | A measure of repetitive behavior frequency. | - Stereotyped behavior - Compulsive behavior - Restricted preferences - Repetitive use of language - Insistence on sameness | Scores calculated by assigning numerical values to Likert scale responses (1 = ‘never or rarely’, 2 = ‘mild or occasional’, 3 = ‘market or notable’, 4 = ‘serious or severe’), with any missing items scored as 1. Raw scores were summed. | Higher scores reflect a greater frequency and/or severity of restricted and repetitive behaviors. | Scores ≥ 3 are interpreted as above a normal level. |
| Flemish Cerebral Visual Impairment Questionnaire (FCVIQ) ^5^ | A measure of behaviors associated with CVI and its impact on everyday functioning. | - Object and face processing impairments - Visual (dis)interest - Clutter and distance viewing impairments - Moving in space impairments - Anxiety-related behaviors | We generated scores for the five factors identified by Ben Itzhak et al. ^6^, scored according to the method described by Crotti et al. ^7^. | Higher scores reflect greater functional visual impairment and a higher likelihood of CVI.  An individual is considered at risk of CVI if they display a minimum of one behavior in at least four of the FCVIQ domains. | Scores are compared to two peer reference groups: (1) children with CVI, and (2) children with both unilateral cerebral palsy and co-occurring CVI. The respective means for each of the five factors are:   - [0.3, 0.23] - [0.325, 0.335] - [0.37, 0.54] - [0.47, 0.61] - [0.3, 0.37] |

## **Supplementary Table 2.** SYT1 gene variant information. Nucleotide and amino acid changes for variants included in the current study are listed along with their classification—complied using the Pathogenicity Evidence tool on Decipher, according to ACMG 2015 criteria ^8,9^. Bolded rows represent new variants not previously reported. All variants are in relation to reference sequence: NM_005639.3.

| **Nucleotide and Amino Acid Change** | | **Domain** | **Reported in previous literature (PMID)** | **ACMG criteria** | **ACMG classification** | **N** |
| --- | --- | --- | --- | --- | --- | --- |
| c.476T>G | p.Leu159Arg(L159R) | C2A | 10, 11 | PM2, PP3, PP2, PS3, PM6 | Likely pathogenic (PP 0.975) | 1 |
| c.551 T>C | p.Val184Ala(V184A) | C2A | 12 | PP3, PP2, PM6 | Uncertain  (PP 0.675) | 1 |
| c.587C>A | p.Thr196Lys(T196K) | C2A | 10, 11 | PM2,  PM3,  PP2,  PS3 | Likely pathogenic (PP 0.949) | 1 |
| c.625G>A | p.Glu209Lys(E209K) | C2A | 10, 11 | PM2, PP3, PP2, PS3, PM6 | Likely pathogenic (PP 0.975) | 1 |
| c.655G>C | p.Glu219Gln (E219Q) | C2A | 10, 11 | PM2, PP3, PP2, PS3, PM6 | Likely pathogenic (PP 0.994) | 1 |
| c.907A>G | p.Met303Val(M303V) | C2B | 10, 11 | PM1, PM2, PP2, PS3,  PM5, PM6 | Pathogenic  (PP 0.999) | 1 |
| c.908T>A | p.Met303Lys (M303K) | C2B | 10, 13 | PM1, PM2, PP2, PS3, PM5, PM6 | Likely pathogenic (PP 0.997) | 1 |
| **c.910G>C** | **p.Asp304His(D304H)** | C2B | - | PM1, PM2, PM5, PP3, PP2, PM6 | Likely pathogenic (PP 0.994) | 1 |
| c.911A>G | p.Asp304Gly(D304G) | C2B | 10, 13, 14 | PM1, PM2, PP3, PS3, PP2, PM6 | Pathogenic  (PP 0.999) | 1 |
| **c.920G>A** | **p.Gly304Asp(G307D)** | C2B | - | PM1, PM2, PM5, PP2, PM6 | Likely pathogenic (PP 0.988) | 1 |
| c.925T>C | p.Ser309Pro(S309P) | C2B | 10, 11 | PM2, PP3, PS3, PP2, PM6 | Pathogenic  (PP 0.994) | 1 |
| **c.926C>T** | **p.Ser309Phe(S309F)** | C2B | - | PM1, PM2, PM5, PM6, PP2, PP3 | Likely pathogenic (PP 0.994) | 1 |
| **c.928G>A** | **p.Asp310Asn(D310N)** | C2B | - | PM1, PM2,  PM6, PP2, PP3 | Likely pathogenic (PP 0.975) | 1 |
| c.930T>A | p.Asp310Glu(D310E) | C2B | 15 | PM1, PM2, PM6, PP2, PP3 | Likely pathogenic (PP 0.975) | 1 |
| **c.935A>G** | **p.Tyr312Cys(Y312C)** | C2B | - | PM1, PM2, PM6, PP2, PP3 | Likely pathogenic (PP 0.975) | 1 |
| **c.989_991delCAA** | **p.Thr330del(T330del)** | C2B | - | PM2, PM4, PM6 | Likely pathogenic (PP 0.9) | 1 |
| c.1022A>G | p.Asn341Ser (N341S) | C2B | 10 | PM2, PM6, PP2, PP3 | Likely pathogenic (PP 0.9) | 1 |
| c.1094A>G | p.Tyr365Cys(Y365C) | C2B | 10, 11 | PM1, PM2, PM6, PS3, PP2, PP3 | Pathogenic  (PP 0.999) | 1 |
| **c.1095_1097dup** | **p.Asp366dup(D366dup)** | C2B | - | PM1, PM2, PM4, PM6 | Likely pathogenic (PP 0.975) | 1 |
| **c.1096G>A** | **p.Asp366Asn(D366N)** | C2B | - | PM1, PM2, PM5, PM6, PP2, PP3 | Likely pathogenic (PP 0.994) | 1 |
| c.1098C>A  c.1098C>G | p.Asp366Glu (D366E) | C2B | 10, 13, 14 | PS1, PS3, PM1, PM2, PM6, PP2, PP3 | Pathogenic (PP 1) | 2  1 |
| c.1100_1102dup | p.Lys367dup(K367dup) | C2B | 10 | PM1, PM2, PM4, PM6 | Likely pathogenic (PP 0.975) | 1 |
| c.1101_1103dup | p.Lys367_Ile368insMet  (K367_I368insM) | C2B duplication, insertion | 16 | PM1, PM2, PM4, PM6 | Likely pathogenic (PP 0.975) | 1 |
| c.1103T​>G | **p.Ile368Ser(I368S)** | C2B | - | PM1, PM2,  PM5, PM6, PP2, PP3 | Likely pathogenic (PP 0.994) | 1 |
| c.1103T>C | p.Ile368Thr(I368T) | C2B | 10, 13,  14, 17, 18 | PM1, PM2, PM6, PP2, PP3, PS3 | Pathogenic  (PP 0.999) | 9 |
| c.1106G>A | p.Gly369Asp(G369D) | C2B | 10, 11 | PM1, PM2, PM6, PP2, PS3 | Pathogenic  (PP 0.997) | 1 |
| c.1113C>G | p.Asn371Lys(N371K) | C2B | 10, 13 | PM1, PM2, PM6, PP2, PS3 | Pathogenic  (PP 0.997) | 2 |
| **c.1199G>A** | **p.Arg400Gln(R400Q)** | Adjacent C2B | - | PP2, PM6 | Uncertain  (PP 0.5) | 1 |
| c.1202C > T | p.Pro401Leu(P401L) | Adjacent C2B | 19 | PM2, PM6, PP2, PS3 | Likely pathogenic  (PP 0.988) | 1 |
| **10 novel variants not previously reported** | | 5 C2A  35 C2B |  |  |  | 40 |

##
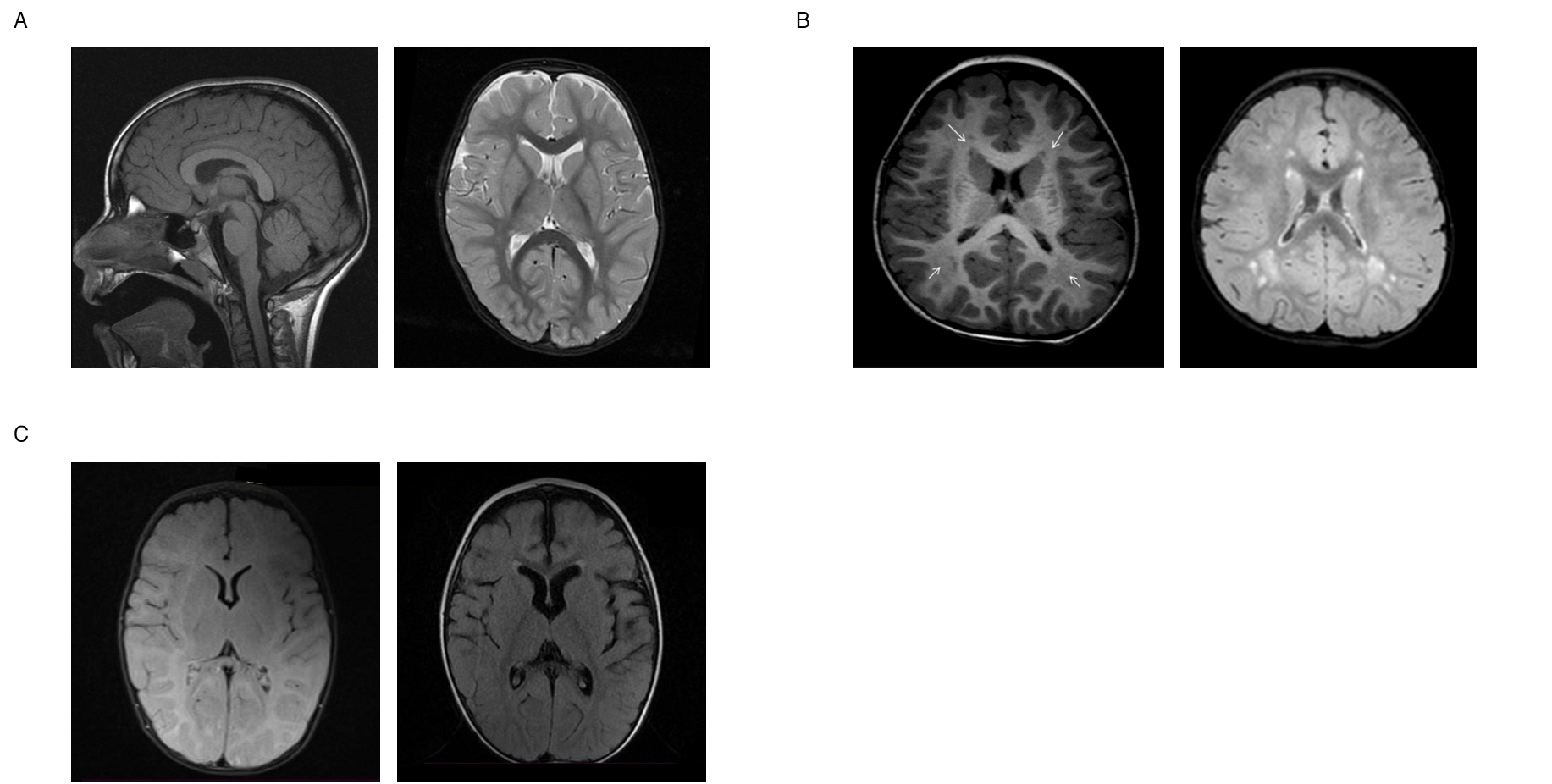

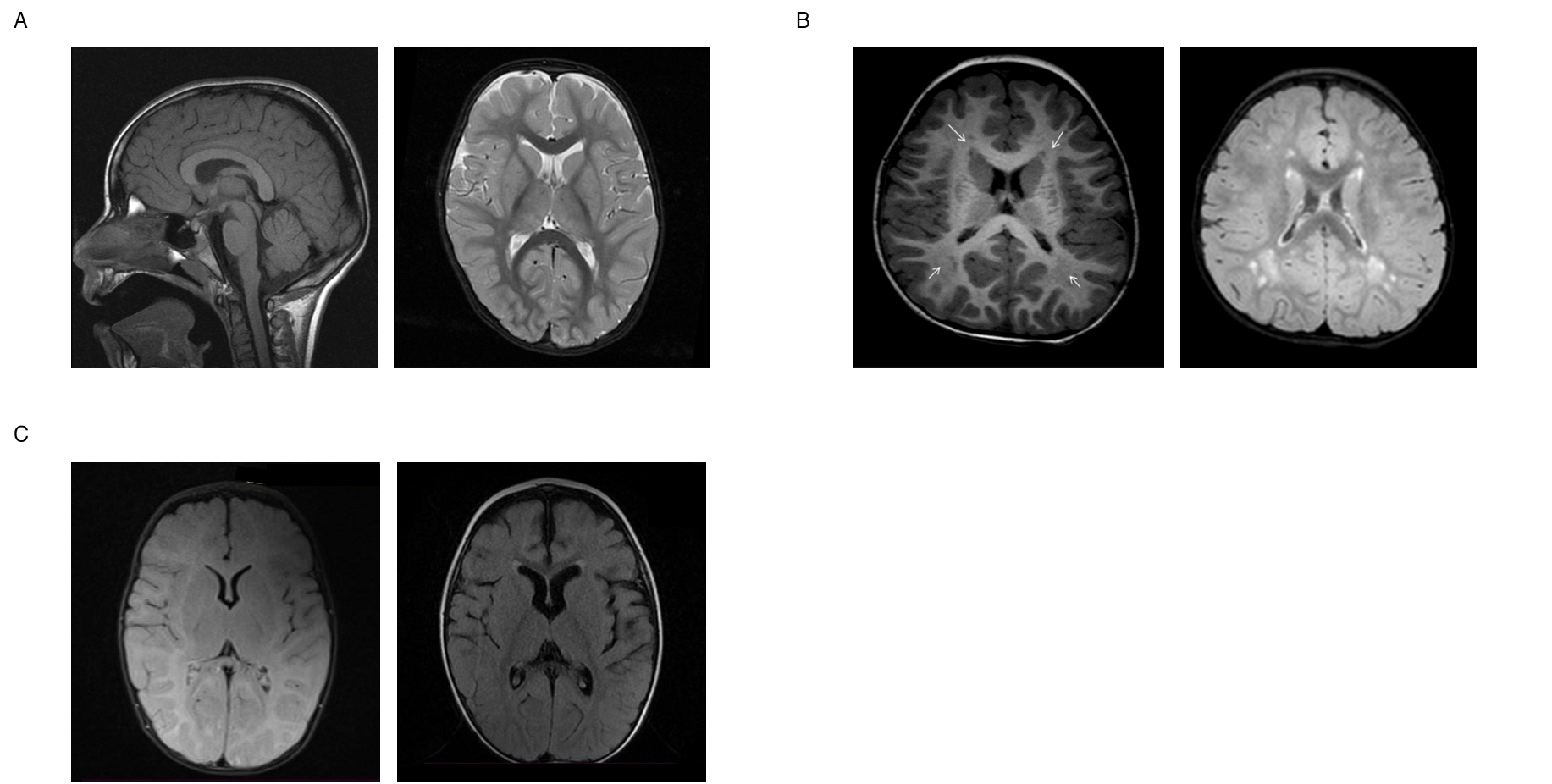
**Supplementary Figure 1.** Sample MRI images.

Serial axial T2 FLAIR MRIs from the same patient at 4 months of age (left) and two-years six-months of age (right) showing deep white matter asymmetry associated with left frontal corico-subcortical atrophy along with secondary dilation of the supratentorial ventricular system.

Axial T1-weighted and FLAIR MRI from two-year one-month old patient displaying atypical periventricular white matter lesions and suspected heterotopia.

Unremarkable axial and sagittal MRI from three-year-old patient.

## **Supplementary Figure 2.** Sample EEG images.


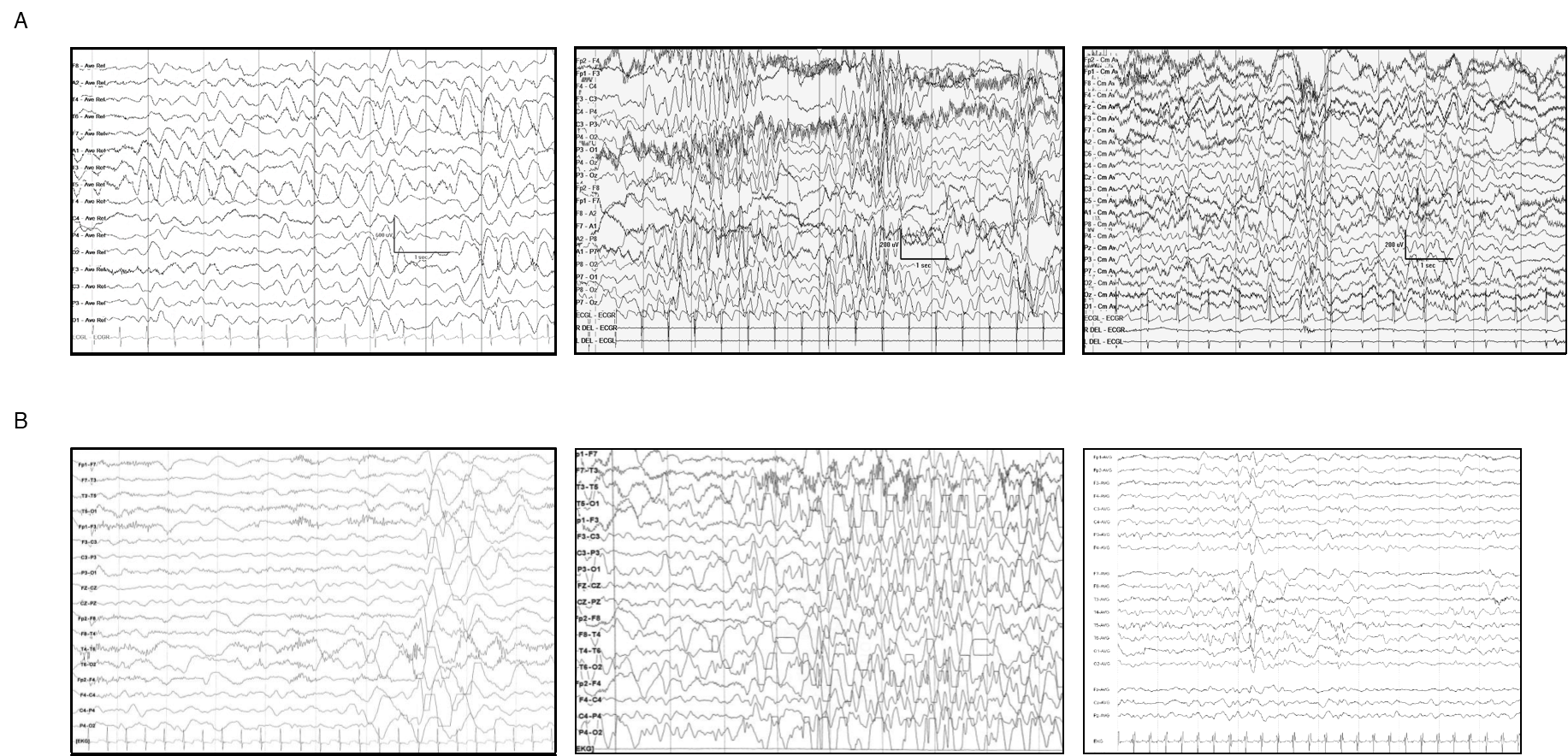


Serial EEGs from the same patient at two, eight, and 11 years of age, showing an evolution in runs of widespread rhythmic theta/delta activities with posterior temporal rhythmic spiking (particularly in the earlier record) and variable associated epileptiform complexes. Left and right subpanels are shown on a common average referential montage; center subpanel on an AP bipolar montage with left-right alternation. Note that the timebase varies across the panels.


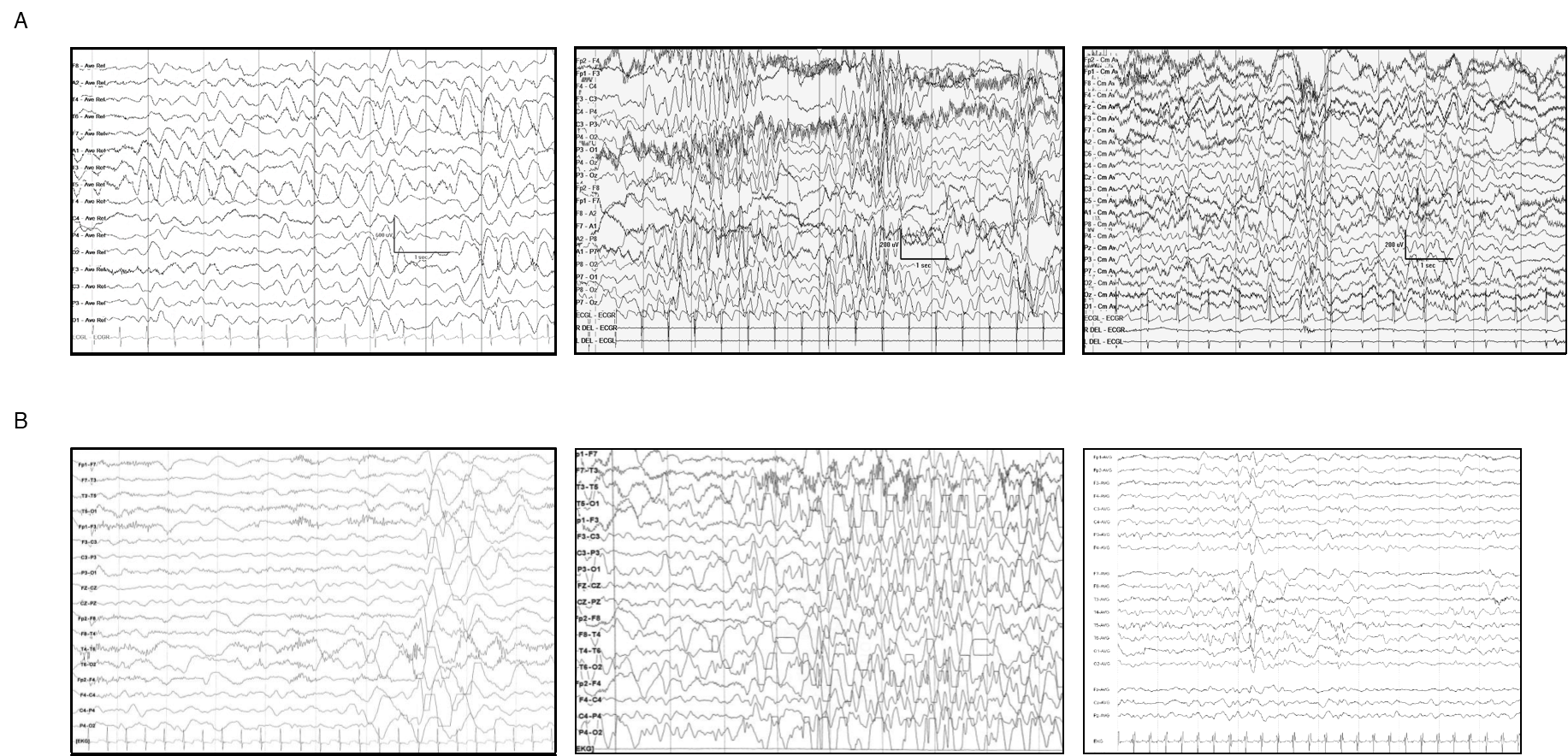


Serial EEGs from the same patient at four months, two years, and 13 years of age displaying high amplitude theta-delta activity with frequent sharp and slow wave complexes. Left and center panel shown on an AP bipolar montage; right subpanel shown on a common average referential montage. Note that the timebase varies across the panels.


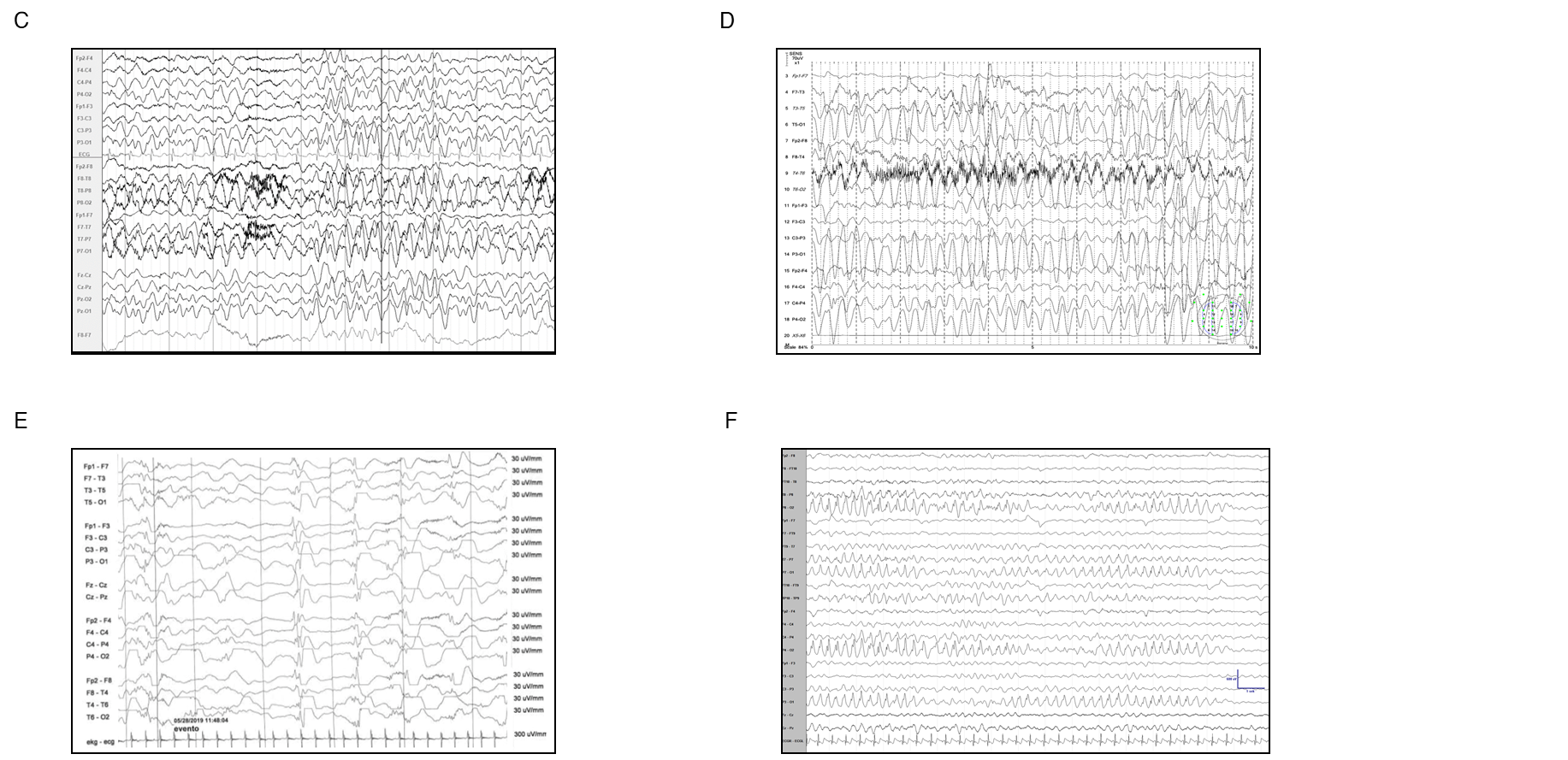


EEG from two-year-old patient displaying high amplitude rhythmic theta/delta (3-4/s) activity predominately in the posterior regions. Shown on an AP bipolar montage.

EEG from two-year eight-month old patient displaying rhythmic slow and sharp waves often in runs of 8-14 sec, with a parieto-temporal maximum (right more than left). Shown on an AP bipolar montage.


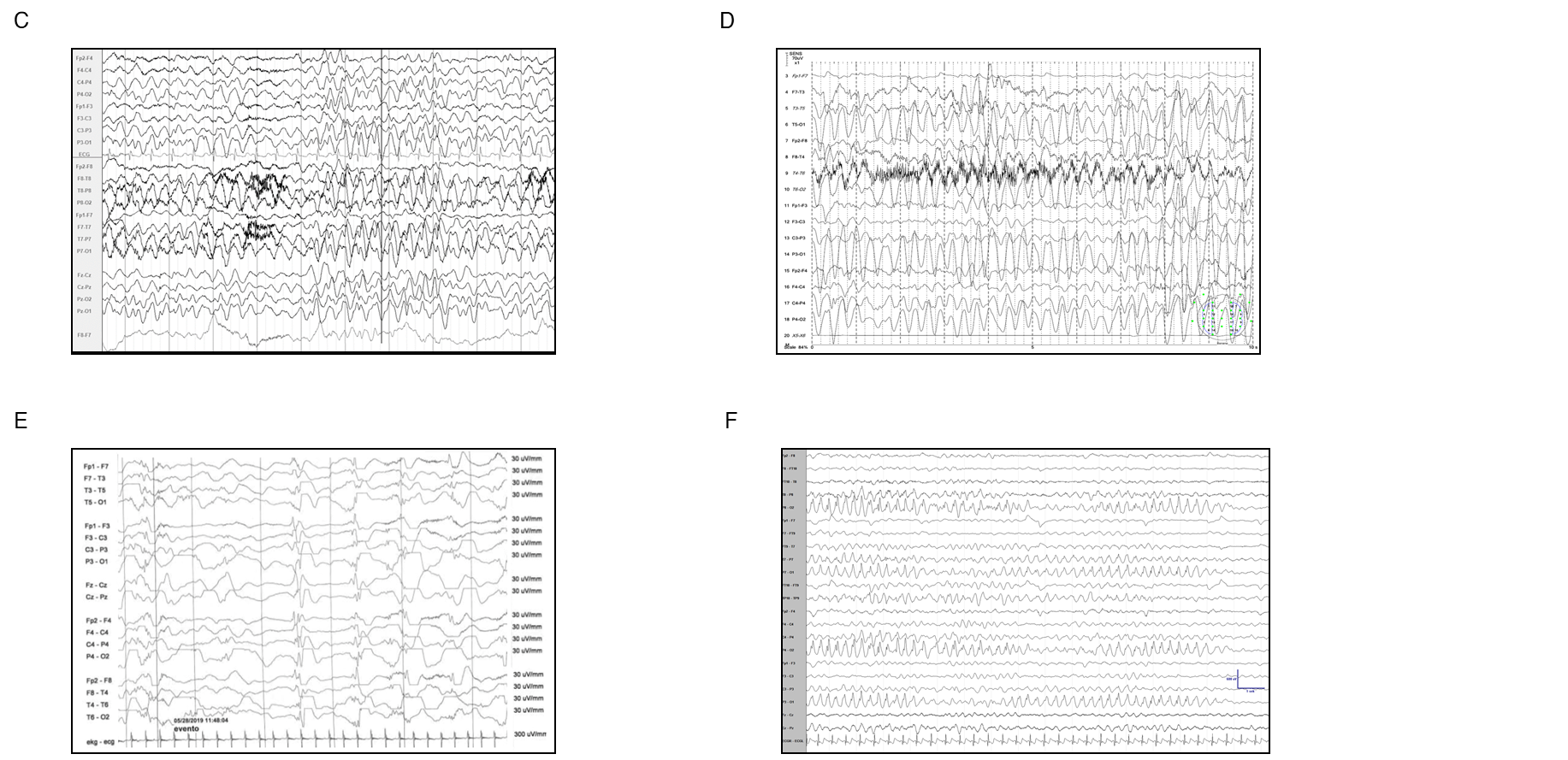


EEG from two-year eight-month old patent displaying a flexor spasm with some electrodecrement. Interictal EEG shows frequent sharp waves/spikes. Shown on an AP bipolar montage.

EEG from three-year-old patient displaying a pattern interpreted as a subclinical seizure including rhythmic spike-wave complexes with posterior maximum. Shown on an AP bipolar montage.


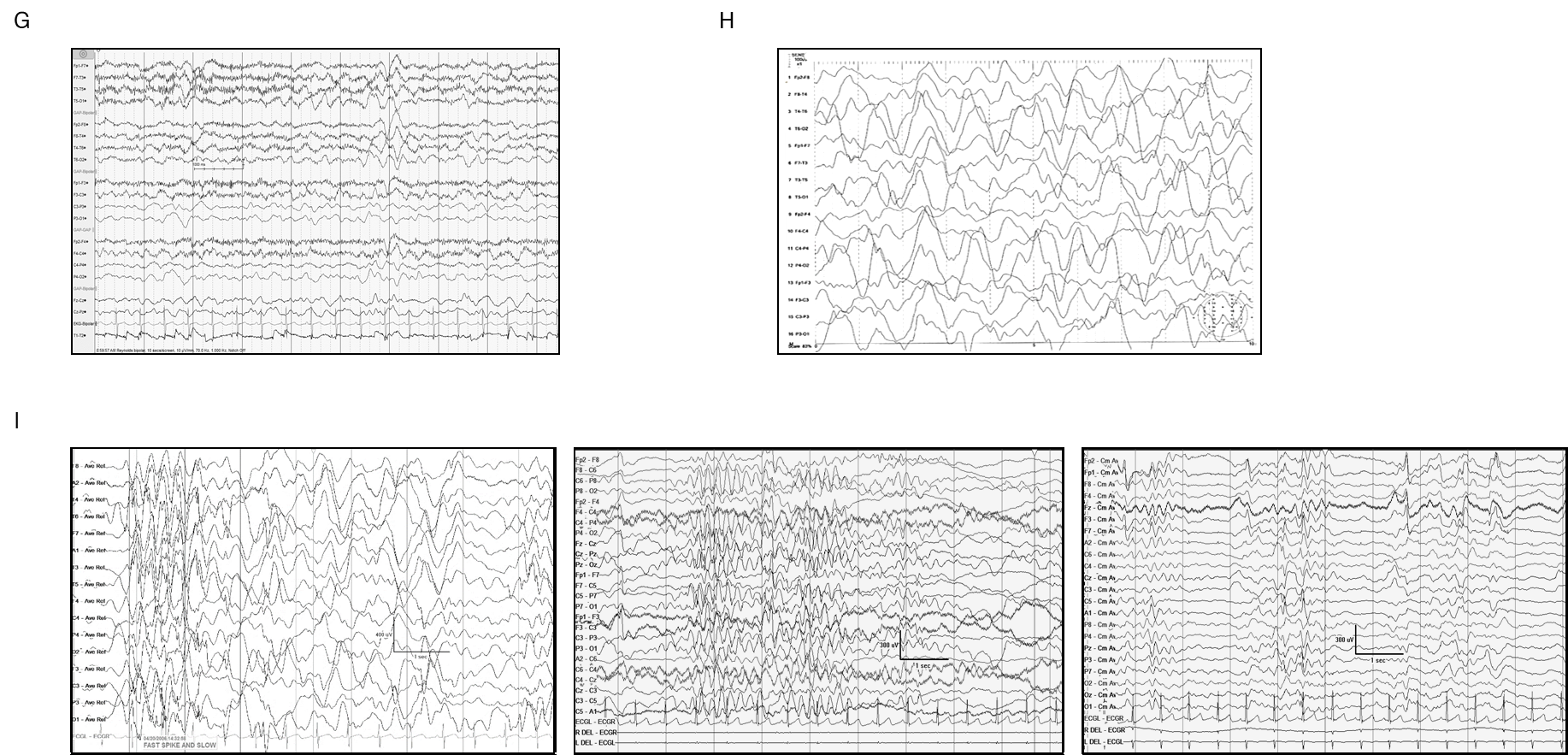


Sleep EEG from eight-month-old patient displaying synchronous high-voltage slow waves and traces of spindles. Shown on an AP bipolar montage.

EEG from three-year old patient displaying some rhythmic delta activity with a posterior maximum and generalized slowing without clear epileptiform activities. Shown on an AP bipolar montage.


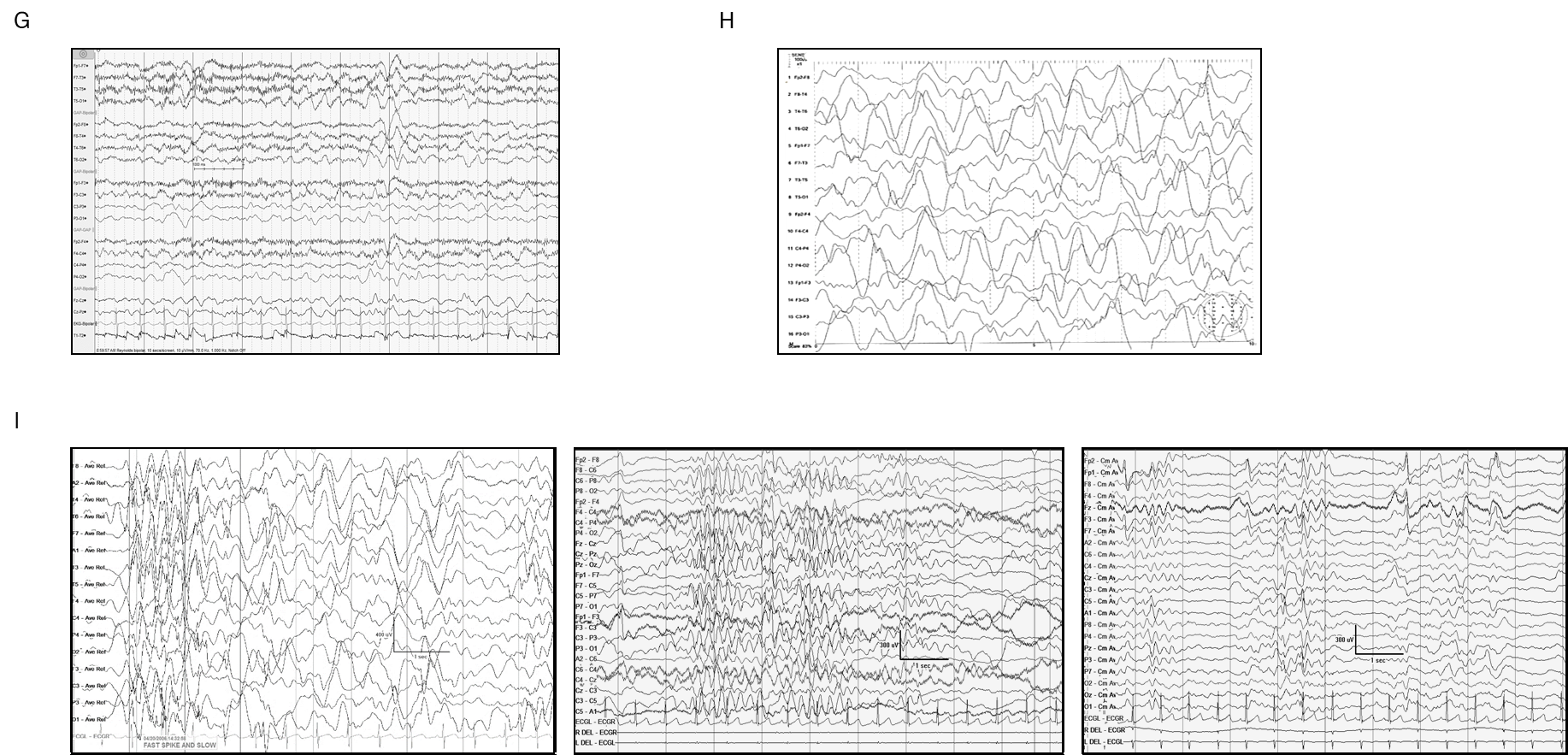


Serial sleep EEGs from the same patient at two, eight, and 11 years of age displaying brief runs of high amplitude fast spike and slow wave complex discharges over fronto-central regions and intermittent generalized bursts of high amplitude theta (5/s) activities. Shown on a common average referential montage (left and right subpanels) or an AP-bipolar montage (center panel). Note subtle differences in timebase between panels.

References

1. Sparrow S, Cicchetti D, Saulnier C. Vineland adaptive behavior scales. Third edition (Vineland-3). Bloomington: NCS Pearson; 2016.

2. Einfeld SL, Tonge BJ. The Developmental Behavior Checklist: The development and validation of an instrument to assess behavioral and emotional disturbance in children and adolescents with mental retardation. J Autism Dev Disord 1995;25(2):81–104.

3. Constantino J, Gruber C. Social Responsiveness Scale. Second Edition (SRS-2). Torrance, CA: Western Psychological Services; 2012.

4. Moss J, Oliver C. The Repetitive Behaviour Scale. Manual for administration and scorer interpretation. Birmingham, UK: University of Birmingham: 2008.

5. Ortibus E, Laenen A, Verhoeven J, et al. Screening for Cerebral Visual Impairment: Value of a CVI Questionnaire. Neuropediatrics 2011;42(04):138–147.

6. Ben Itzhak N, Vancleef K, Franki I, et al. Visuoperceptual profiles of children using the Flemish cerebral visual impairment questionnaire. Dev Med Child Neurol 2020;62(8):969–976.

7. Crotti M, Ortibus E, Mailleux L, et al. Visual, perceptual functions, and functional vision in children with unilateral cerebral palsy compared to children with neurotypical development. Dev Med Child Neurol 2024;66(8):1084–1095.

8. Bragin E, Chatzimichali EA, Wright CF, et al. DECIPHER: database for the

interpretation of phenotype-linked plausibly pathogenic sequence and copy-number variation. Nucleic Acids Res. 2014;42(Database issue):D993–D1000.

9. Richards S, Aziz N, Bale S, et al. Standards and guidelines for the interpretation of sequence variants: a joint consensus recommendation of the American College of Medical Genetics and Genomics and the Association for Molecular Pathology. Genet Med 2015;17(5):405–424.

10. Melland H, Bumbak F, Kolesnik-Taylor A, et al. Expanding the genotype and phenotype spectrum of SYT1-associated neurodevelopmental disorder. Genet Med 2022;24(4):880–893.

11. Park PY, Bleakley LE, Saraya N, et al. Correlation between evoked neurotransmitter release and adaptive functions in SYT1-associated neurodevelopmental disorder. EBioMedicine 2024;109:105416.

12. Huang W, Yang Y, Che F, et al. Lethal variant in the C2A domain may cause severe

SYT1-associated neurodevelopmental disorder in the newborns. Neurogenetics 2024;25(1):27–31.

13. Baker K, Gordon SL, Melland H, et al. SYT1-associated neurodevelopmental disorder: a case series. Brain 2018;141(9):2576–2591.

14. Bradberry MM, Courtney NA, Dominguez MJ, et al. Molecular Basis for Synaptotagmin-

1-Associated Neurodevelopmental Disorder. Neuron 2020;107(1):52-64.e7.

15. Cotrina-Vinagre FJ, Rodríguez-García ME, Pozo-Filíu LD, et al. Expanding the genetic

and phenotypic spectrum of Baker-Gordon syndrome: a new de novo SYT1 variant. J Genet 2024;103:24.

16. Cesaroni CA, Spagnoli C, Baga M, et al. Expanding Phenotype of SYT1-Related

Neurodevelopmental Disorder: Case Report and Literature Review. Mol Syndromol 2023;14(6):493–497.

17. Baker K, Gordon SL, Grozeva D, et al. Identification of a human synaptotagmin-1

mutation that perturbs synaptic vesicle cycling. J Clin Invest 2015;125(4):1670–1678.

18. Porto MB, Castro G da ME, Pereira SSS, et al. c.1103T>C (p.Ile368Th) de novo Variant

in Synaptotagmin 1 (SYT1) Gene is Pathogenic, Leading to an Ultra-Rare Neurodevelopmental Disorder: The Baker-Gordon Syndrome. Int Med Case Rep J 2024;17:63–70.

19. van Boven MA, Mestroni M, Zwijnenburg PJG, et al. A de novo missense mutation in

synaptotagmin-1 associated with neurodevelopmental disorder desynchronizes neurotransmitter release. Mol Psychiatry 2024;29(6):1798–1809.
